# Facial Soft Tissue Thickness of Midline in an Iranian Sample: MRI Study

**DOI:** 10.2174/1874210601711010375

**Published:** 2017-06-30

**Authors:** Masume Johari, Farzad Esmaeili, Hadi Hamidi

**Affiliations:** 1 Oral Radiology, Dental and Periodontal Research Center, Tabriz University of Medical Sciences, Tabriz, Iran; 2 Oral Radiology, Baghiatallah University, Tehran, Iran

**Keywords:** Anatomic landmark, Magnetic Resonance Imaging, Soft tissue thickness, Midface, Iranian

## Abstract

**Background and Aim:**

To identify human skeletal remains, different methods can be used and using these techniques, important data can be obtained. However, facial reconstruction is the last method to indentify unknown human faces which requires knowledge about facial soft tissue thickness in the different positions of the face. The present study determined the facial soft tissue thickness in the different landmark points on the MRI images of patients referred to Radiology Department of Shahid Madani Hospital.

**Materials and Methods:**

In this descriptive cross-sectional trial, MRI images of 179 patients (61 males, 118 females) in the age range of 18-76 years old who did not show any pathologic lesions, were selected. The measurements of the facial soft tissue were done on 12 landmark points on the midline area by two radiologist observers using specific software on the images. The differences in the soft tissue thickness in these landmark points were statistically analyzed by Mann-Whitney U (in term of gender) and Kruskal-Wallis tests (in terms of Body Mass Index [BMI] and age groups). P value less than 0.05 was considered statistically significant. The data were compared with the results of other studies.

**Results:**

The results obtained in the present study were higher than Turkish and American studies in most of the landmark points. Facial soft tissue thickness in most of the landmarks was more in males than females. In some of the landmarks, significant differences were found between emaciated, normal and overweight patients while in most cases, soft tissue thickness increased with the increased BMI. In some cases, significant differences were noted between soft tissue thickness values among the different age groups, in which the thickness increased or thinned with the increased age.

**Statistical Analysis:**

There were statistically significant associations between the presence and surface area of Haller cells and the occurrence of ipsilateral maxillary sinusitis. Neither the angulation of the uncinate process nor the size of the maxillary sinus ostium significantly correlates with the formation of maxillary sinusitis.

**Conclusion:**

The data achieved in the present study can be used for the facial reconstruction purposes in the Iranian population; however, the slight differences existing between the studied population and other subgroup races must be considered for accurate reconstructions.

## INTRODUCTION

It is important to identify a skeletonized cadaver. In these cases, facial features are distorted so identity of the deceased is unrecognizable [1]. DNA analysis, dental records and finger prints methods are dependent on comparative databases [2]. Characteristics such as age, sex, race, and body size cannot be used without comparison with ante mortem information [3]. Facial reconstruction technique is the best way to identify a person in a situation that other techniques are useless [4]. To reconstruct the face accurately, further information such as hair color, eyelid, ear shape and facial soft tissue thickness is needed [4]. Facial soft tissue thickness is necessary for facial reconstruction because all other features have been lost or damaged [5]. Previous studies showed that there is a significant variation in facial tissue thickness between different populations. Databases of facial soft tissue thickness are available for Australian, Brazilian, Japanese, Portuguese, Egyptian, Indian, Turkish and Slovak [1,3-12]. El-Mehallawi and Soliman found out that there is a significant difference in facial thickness of different genders [13]. Also different body types (overweight, normal, emaciated) play important roles in facial reconstruction [14-16].

Determining offset of soft tissue at specific landmarks is the principle of facial reconstruction [1]. There are different methods established to determine soft tissue thickness, *e.g.* puncture, x-ray imaging, CT images, CBCT images, ultrasonic and MRI images [4, 5, 12, 13,17]. Each of these methods has its own limitations [13, 18-20]. Recently magnetic resonance imaging has been used mostly because of its accuracy and hazardless characteristics [1, 2, 21].

As we know, there is no database of facial soft tissue thickness among Iranian population in relation to sex, age and BMI and this lake of information makes it difficult to reconstruct facial soft tissue. The aim of this study was to collect a database of average facial soft tissue thickness measured on MRI in relation to sex, age and BMI for the Iranian population. This article represents the first database of facial soft tissue thickness for Middle East countries.

## MATERIALS AND METHODS

This descriptive cross-sectional study included 179 patients (60 males and 119 females) between the ages of 11 and 76 years old. The patients were candidates for brain MRI in the radiology clinic during December 2012 and March 2013. The MR images were required for diagnostic purposes. All edentulous patients and those with trauma, known systemic disease, previous history of surgery, soft tissue and bone masses and those taking medications that influence the thickness of soft tissue like Cortisol, or any other pathology that may distort the soft tissue thickness excluded from the study.

**Fig. (1):**
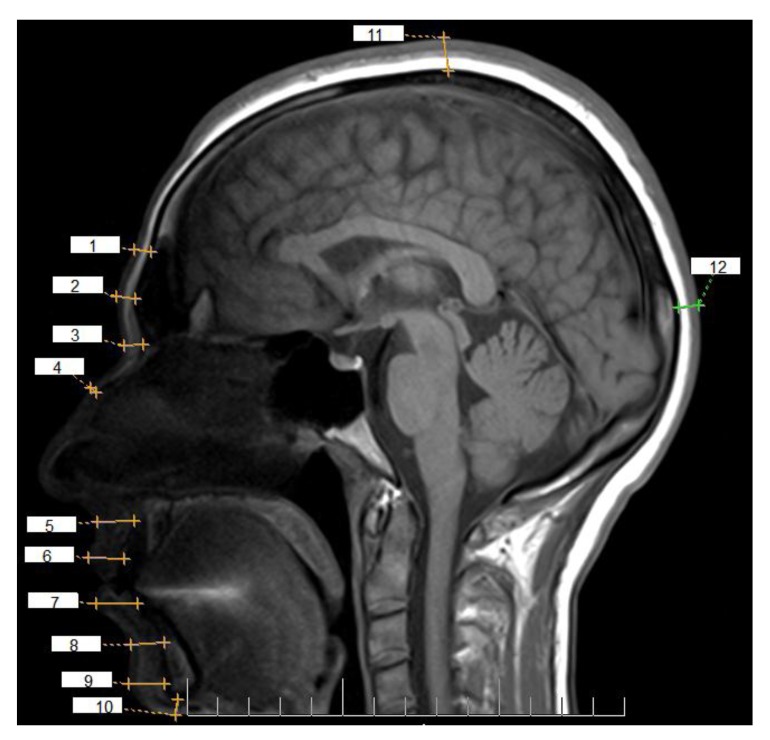
Anatomical landmarks: (1) Supraglabella. (2) Glabella. (3) Nasion. (4) End of nasal. (5) Mid-philtrum .(6)Upper lipmargin. (7)Lower lip margin. (8) Chin lip fold. (9) Mental eminence. (10) Beneath chin. (11) Vertex. (12) Opisthocranion.

Patient’s information such as sex, age, weight and height was collected before the examination. Body mass index was calculated by placing the height and weight at the formula (kg/m^2^). Siemens magnetom avanto 1.5 Tesla unit were used to performing images. The T1-weighted sagittal images were used to measure 12 landmarks placed at midsagittal according to the studies of Sahni *et al.*, Penekova *et al.* and Spahioghlu *et al.* [1-3]. Landmarks consist: opisthocranion (op), vertex (v), supraglabella (sg), glabella(g), nasion(n), end of nasal(end), mid-philtrum(mid), upper lip margin(ul),lower lip margin(ll), chin lip fold(clf), mental eminence(me) and beneath chin(bc) (Fig. **1**).

The fusion eFilm 2.1.2 software was used to measure soft tissue thickness. All collected data were analyzed using SPSS 21 software. A test was performed for Intra-observer errors by repeating measurements for 20 cases. The mean, standard deviation (SD) and range were calculated for all the landmarks. Data were classified by differences between age, sex, and BMI. The data were compared with other studies and the differences were noted. Independent t-test and one-way analysis of variance (ANOVA) were used as statistical analysis.

## RESULTS

Our study demonstrates that there was a significant difference between men and women in the age range of 18-34 years old, according to some landmarks including nasion, end of nasal, mid-philtrum, upper lip margin, lower lip margin, chin-lip fold, mental eminence, and beneath chin; and soft tissue thickness in men was significantly more than women. In other variables, there was no significant difference between two groups (Table **1**).

Also, in the age range of 35-45 years old, some thickness of soft tissue landmarks including nasion and upper lip margin, were significantly higher in men. In other landmarks, there was no significant difference between two genders (Table **1**).

Furthermore, in age range of >46 years old, soft tissue thickness in landmarks of mid-philtrum, upper lip margin, lower lip margin, chin-lip fold, and opisthocranion were significantly higher in men. However, in other landmarks, there was no significant difference between the two groups (Table **1**).

Comparison of the landmarks in men by age groups showed that there was a significant difference in 3 groups’ (18-34, 35-45, and >46 years old) soft tissue landmarks including mid-philtrum, but there was not a significant difference in other landmarks (Table **2**).

As well as, there was a significant difference between two age groups of 18-34 and ≥ 46 years old in mid-philtrum. Also, a significant difference between two age groups of 18-34 and 35-45 years old was found in mid-philtrum. In addition, there was no significant difference between two groups of 35-45 and ≥ 46 years old in mid-philtrum (Table **2**).

Comparison of the landmarks in women by age groups showed that there was a significant difference in 3 groups’ (18-34, 35-45, and >46 years old) soft tissue landmarks including upper lip margin, but there was no significant difference in other landmarks (Table **2**).

There was a significant difference between two age groups (18-34 and 35-45 years old) in upper lip margin (Table **2**).

Also, there was a significant difference between two age groups (18-34 and ≥ 46 years old) in upper lip margin (Table ** 2**).

In addition, no significant difference was found in two age groups (35-45 and ≥ 46 years old) in upper lip margin (Table **1**).

Comparison of the landmarks in men by weight groups (low weight, normal weight, overweight) showed that there was a significant difference in three groups in some of the landmarks including mental eminence and vertex. Nevertheless, there was no significant difference between groups in other landmarks (Table **3**).

Our results showed a significant difference between low weight and normal weight samples in vertex, but the difference between two groups in landmark of mental eminence was not significant (Table **3**).

Also, there was a significant difference between low weight and overweight samples in vertex and mental eminence (Table **3**).

Nevertheless, there was no significant difference between two groups in Mental eminence and vertex (Table **3**).

On the other hand, comparison of the landmarks in women by weight groups (low weight, normal weight, overweight) showed that there was a significant difference in three groups in amount of soft tissue thickness in glabella, nasion, end of nasal, mental eminence, and beneath chin (Table **3**).

Result of comparison between low weight and normal weight women demonstrated that there was a significant difference between two groups in amount of nasion, but no significant difference was found between two groups in amount of glabella, end of nasal, mental eminence, and beneath chin (Table **2**).

There was no significant difference between two groups (low weight and overweight samples) in amount of glabella and end of nasal landmarks, but the difference was significant in amount of nasion, mental eminence, and beneath chin (Table **3**).

Also, in normal weight and overweight groups, a significant difference was found in all the landmarks including glabella, nasion, end of nasal, mental eminence, and beneath chin (Table **3**).

## DISCUSSION

Due to the lack of soft tissue thickness in the Iranian samples in the present study, soft tissue thickness quantities ​​were measured in a group of patients in the Northwest of Iran. For this purpose, T1-weighted images of patients who underwent MRI evaluations for various reasons, were evaluated in sagittal slices.

So far, few studies have been carried out using MRI [1, 3, 21]. There is no accessibility to a large number of samples using methods such as CT and X-Ray radiation because of too much radiation; thus, similar studies have been conducted on small number of patients [18-20]. Ultrasonic method due to being operator dependent and probe pressure on tissue is not accurate.

MRI is a radiological imaging method for observing internal structures of the human being. In this method, in addition to detailed anatomical structures, soft tissue details are available in different planes which prove its advantage, in comparison with other methods. Yet it should be noted that this method is expensive than other methods; therefore, possibility of its use, for purposes of determining the thickness of the soft tissue, is limited in some cases. However, the sagittal and axial slices of tissue from the midline and midface MRI, performed at the hospital for patients, is used for the preparation of the database of related soft tissue thickness.

In a similar study, mid-line landmarks were measured [2, 3]. In this study, mid-line landmarks were measured too, in Iranian population. Mid-face is the most important region of face for recognition, and in this regard mid-line has special importance [22].

In this study, patient’s MRI measurement was performed in the supine position like Sipahioglu and colleagues’ study [3]. Some studies demonstrated gravity in supine and standing position has a different influence on some face thickness [18, 23, 24]. Also, some research have shown that soft tissue thickness varies in different ethnic groups [6, 13, 21, 22].

Shepherd and colleagues demonstrated that gender and race have important role in face recognition. Therefore, each gender’s data should be used on face recognition accuracy of the same gender [25]. In this study, there was a significant difference in some soft tissue thickness landmarks by gender and age groups.

However, in the study by Stephen and Domaracki, it was shown that there was no significant difference between soft tissue thickness of Australian men and women [6].

Sahni and colleagues demonstrated facial soft tissue thickness in men was more than women in Northwest India [1]. Also, Dong and colleagues reported, in most cases, the anthropological landmarks in men were more than women. In our study, similar results were obtained [14].

Our study showed, in most cases with BMI rise, soft tissue thickness increased in most landmarks significantly. Also some study has shown a significant relationship between BMI and soft tissue thickness [3].

Ward and Starbuck could determine weight effects on face recognition accuracy by obese, normal and thin faces reconstruction in a skull and using data of soft tissue thickness [26].

Using BMI changes, Sahni and colleagues demonstrated that the face change in men was more obvious as compared to women.

According to our study, facial soft tissue thickness change, based on body composition, has minimal effects on facial form detection results; however, it influences the detection manner significantly.

Wilkinson and colleagues showed soft tissue thickness decreases in mouth and chin but increases around the eyes with aging [27].

Panenkova and colleagues reported that in women, soft tissue thickness in mid-philtrum decreased with aging but there was no significant difference in chin landmarks. As well as, our study demonstrated that there was no significant relationship between soft tissue thickness decrease in mid-philtrum in women and aging [2].

De Greef and colleagues’ study have shown soft tissue thickness around mouth landmarks including mid-philtrum, upper lip margin, nasio-labial ridge, and supracanina, decreased approximately 1-2 mm by aging [18].

Our study showed that, soft tissue thickness in mid-philtrum, lower lip and upper lip landmarks were thicker in men compared to women in all age groups except mid-philtrum in age group of 35-45 years old. It means that the lips of men were thicker than women. Similar results were reported in Korean adult [28].

## CONCLUSION

Totally, the data achieved in the present study can be used for facial reconstruction purposes in the Iranian population; however, the slight differences existing between the studied population and other subgroup races must be considered for accurate reconstructions.

## Figures and Tables

**Table 1:** Comparison of males and females (a) between 18 and 24 years, (b) between 25 and 45 years and (c) over 46 years (all the measurements in millimeter).

Landmarks	**Male**					**Female**					***P* Value**
**(A)**	**Mean**	**S.D.**	**Min**	**Max**	**Rang**	**Mean**	**S.D.**	**Min**	**Max**	**Rang**	
Supraglabella	5.07	1.13	3	8	5	4.73	1.14	2	9	7	0.23
Glabella	6.66	1.34	4	10	6	6.86	1.31	4	10	6	0.41
Nasion	8.41	1.8	5	11	6	7.38	1.78	3	13	10	0.009
End of nasal	3.28	2.28	2	13	11	2.48	0.69	1	4	3	0.04
Mid-philtrum	16.41	3.19	11	23	12	13.95	2.55	6	22	16	0.001
Upper lipmargin	13.86	2.13	10	17	7	11.86	1.93	8	17	9	0.0001
Lower lip margin	14.62	1.93	10	18	8	13.17	2.03	6	17	11	0.002
Chin lip fold	12.24	1.64	9	16	7	11.41	1.92	6	16	10	0.05
Mental eminence	15.38	3.89	10	28	18	12.75	2.21	9	17	8	0.001
Beneath chin	8.64	1.99	5	12	7	7.24	1.62	5	12	7	0.003
Vertex	7.14	1.43	5	10	5	7.31	1.72	2	11	9	0.58
Opisthocranion	6.18	1.25	4	8	4	5.97	1.57	3	12	9	0.43
**(B)**											
Supraglabella	5.2	1.62	3	9	6	4.25	1.29	2	7	5	0.13
Glabella	7.8	1.75	5	11	6	7.35	1.39	5	11	6	0.59
Nasion	9	1.05	7	10	3	7.6	1.73	5	12	7	0.02
End of nasal	3	0.82	2	5		2.6	0.59	1	3	2	0.33
Mid-philtrum	12.7	4.29	2	17	15	13.4	1.27	11	16	5	0.98
Upper lipmargin	12.2	1.93	9	16	7	10.6	1.39	9	14	5	0.02
Lower lip margin	14.3	2	11	17	6	13.1	2.07	9	17	8	0.18
Chin lip fold	12.2	1.48	10	15	5	11.7	1.92	9	16	7	0.5
Mental eminence	15.2	3.68	11	22	11	12.6	2.3	7	17	10	0.07
Beneath chin	9	2.55	5	14	9	7.47	1.74	5	11	6	0.09
Vertex	7.78	1.86	6	12	6	6.85	2.03	3	12	9	0.22
Opisthocranion	6.11	1.17	4	8	4	6.15	1.35	4	9	5	0.95
(C)											
Supraglabella	5	0.77	4	6	2	4.96	1.07	3	7	4	0.97
Glabella	6.78	1.35	4	10	6	7.11	1.26	4	9	5	0.29
Nasion	8.89	1.84	6	13	7	8.29	1.8	5	13	8	0.37
End of nasal	2.89	0.58	2	4	2	2.71	0.53	2	4	2	0.32
Mid-philtrum	14.11	3.66	2	19	17	13.14	4.79	7	33	26	0.04
Upper lipmargin	12.67	2.63	7	18	11	10.67	1.92	7	14	7	0.007
Lower lip margin	14.28	2.14	11	18	7	12.54	2.08	8	16	8	0.02
Chin lip fold	12.61	1.54	9	15	6	11.25	1.43	7	15	8	0.004
Mental eminence	14.39	2.19	11	19	8	13.68	2.07	10	18	8	0.34
Beneath chin	8.5	2.47	5	13	8	7.86	1.82	4	12	8	0.44
Vertex	6.35	1.37	3	8	5	6.68	1.52	3	10	7	0.49
Opisthocranion	6.67	1.75	2	9	7	5.85	1.18	4	8	4	0.04

R, Right; L, left.

*p* < 0.05.

**Table 2:** Age comparison for: (a) males and (b) females (all the measurements in millimeter).

	**18-24**		**25-45**		**>46**		***P* Value**
**Landmarks**	**Mean**	**S.D.**	**Mean**	**S.D**	**Mean**	**S.D.**	
**(a)**							
Supraglabella	5.07	1.13	5.2	1.62	5	0.77	0.99
Glabella	6.66	1.34	7.8	1.75	6.78	1.35	0.14
Nasion	8.41	1.8	9	1.05	8.89	1.84	0.63
End of nasal	3.28	2.28	3	0.82	2.89	0.58	0.81
Mid-philtrum	16.41	3.19	12.7	4.29	14.11	3.66	0.02
Upper lip margin	13.86	2.13	12.2	1.93	12.67	2.63	0.09
Lower lip margin	14.62	1.93	14.3	2	14.28	2.14	0.75
Chin lip fold	12.24	1.64	12.2	1.48	12.61	1.54	0.62
Mental eminence	15.38	3.89	15.2	3.68	14.39	2.19	0.77
Beneath chin	8.64	1.99	9	2.55	8.5	2.47	0.89
Vertex	7.14	1.43	7.78	1.86	6.35	1.37	0.13
Opisthocranion	6.18	1.25	6.11	1.17	6.67	1.75	0.33
**(b)**	**Mean**	**S.D.**	**Mean**	**S.D**	**Mean**	**S.D.**	
Supraglabella	4.73	1.14	4.25	1.29	4.96	1.07	0.09
Glabella	6.86	1.31	7.35	1.39	7.11	1.26	0.25
Nasion	7.38	1.78	7.6	1.73	8.29	1.8	0.09
End of nasal	2.48	0.69	2.6	0.59	2.71	0.53	0.16
Mid-philtrum	13.95	2.55	13.4	1.27	13.14	4.79	0.09
Upper lip margin	11.86	1.93	10.6	1.39	10.67	1.92	0.006
Lower lip margin	13.17	2.03	13.1	2.07	12.54	2.08	0.37
Chin lip fold	11.41	1.92	11.7	1.92	11.25	1.43	0.79
Mental eminence	12.75	2.21	12.6	2.3	13.68	2.07	0.17
Beneath chin	7.24	1.62	7.47	1.74	7.86	1.82	0.31
Vertex	7.31	1.72	6.85	2.03	6.68	1.52	0.12
Opisthocranion	5.97	1.57	6.15	1.35	5.85	1.18	0.81

R, Right; L, left.

*p* < 0.05.

**Table 3:** Tissue depth means of BMI subgroups of (a) males and (b) females (measurements in millimeter).

**Landmark**	**BMI < 20**		**BMI 21– 25**		**BMI > 26**		***P* Value**
**(a)**	**Mean**	**S.D.**	**Mean**	**S.D.**	**Mean**	**S.D.**	
Supraglabella	4.67	0.82	4.82	1.07	5	0.83	0.69
Glabella	6.33	0.83	6.55	1.04	7.33	1.43	0.18
Nasion	8.5	1.97	8.64	1.8	8.75	1.42	0.92
End of nasal	2.83	0.75	3.18	2.04	2.88	0.61	0.81
Mid-philtrum	16	3.03	14.18	2.44	14.71	4.96	0.54
Upper lipmargin	13.67	1.21	12.82	2.64	13.38	2.34	0.83
Lower lip margin	13.33	1.75	15	1.95	14.63	1.61	0.17
Chin lip fold	11.67	1.86	11.91	1.87	12.46	1.56	0.65
Mental eminence	11.83	2.48	13.89	3.41	15.39	3.64	0.05
Beneath chin	6.33	1.15	7.44	2.35	8.65	2.41	0.14
Vertex	5.67	0.82	6.73	1.42	7.39	1.27	0.01
Opisthocranion	5.33	1.03	5.28	1.83	6.5	1.35	0.13
	**BMI <20**		**BMI 21–25**		**BMI >26**		***P* Value**
**(b)**	**Mean**	**S.D.**	**Mean**	**S.D.**	**Mean**	**S.D.**	
Supraglabella	4.71	1.79	4.55	1.18	4.78	1.17	0.42
Glabella	6.71	1.38	6.74	1.64	7.5	1.15	0.01
Nasion	5.57	1.62	7.21	1.89	8.25	1.68	0.001
End of nasal	2.29	0.49	2.42	0.68	2.68	0.57	0.05
Mid-philtrum	15	2.08	13.79	2.09	12.9	2.42	0.09
Upper lipmargin	12	2.38	11.53	1.93	10.9	1.78	0.43
Lower lip margin	12.57	1.9	13.8	2.03	12.9	1.98	0.72
Chin lip fold	11.86	1.46	11.11	1.75	11.46	1.9	0.43
Mental eminence	11.29	2.29	12.39	2.22	13.41	2.47	0.05
Beneath chin	6.43	0.79	7.09	1.58	8.06	1.88	0.03
Vertex	7	0.82	6.95	1.9	6.83	1.78	0.93
Opisthocranion	5.43	1.9	5.63	1.26	6.15	1.41	0.28

R, Right; L, left.

*p* <0.05.
